# Artificial Intelligence–Enabled Serial Electrocardiograms for Prediction of All-Cause Mortality in Secondary Care Settings

**DOI:** 10.1016/j.jacadv.2026.102875

**Published:** 2026-06-17

**Authors:** Gal Tsaban, Asaf Harari, Adi Shiloh, David Shamia, Lior Rokach, Michal Gordon, Moti Haim, Gilad Katz

**Affiliations:** a Cardiology Department, Soroka University Medical Center, Beersheva, Israel; b Faculty of Health Sciences, Ben Gurion University of the Negev, Beersheva, Israel; c Faculty of Computer and Information Science, Ben Gurion University of the Negev, Beersheva, Israel; d Clinical Research Center, Soroka University Medical Center, Beersheva, Israel

**Keywords:** all-cause mortality, artificial intelligence-enabled electrocardiogram, prediction, secondary prevention settings

## Abstract

**Background:**

Prognostic assessment in secondary care settings remains challenging and may influence clinical decision-making and follow-up. Artificial intelligence–enabled electrocardiography (AI-ECG) is a promising tool, but the value of incorporating serial ECG data remains unclear.

**Objectives:**

This study aimed to develop and validate an AI-ECG model for predicting 1-year all-cause mortality and to assess the incremental value of serial ECGs.

**Methods:**

We conducted a retrospective cohort study of adults aged over 50 years treated in emergency department or inpatient settings between 2014 and 2019. Models were developed using a deep-learning framework applied to either a single index ECG or serial ECGs, including the index and 2 prior recordings. Data were divided into training, validation, and test sets. Performance was assessed using discrimination, calibration, and decision-curve analysis. A classification threshold was selected on the validation set by maximizing the F1 score and applied to the test set.

**Results:**

A total of 13,417 patients were included (median age 69 years [IQR: 57-80]; 45% women), of whom 4,372 (34%) died within 1 year. The serial AI-ECG model achieved an area under the curve of 0.84 (95% CI: 0.824-0.862). At a threshold of 0.24, sensitivity was 70%, specificity was 80%, and positive predictive value was 49%. Calibration showed good agreement between predicted and observed risks (Brier score: 0.123). Decision-curve analysis demonstrated greater net benefit than treat-all and treat-none strategies. Addition of clinical variables beyond age and sex did not improve performance.

**Conclusions:**

A serial AI-ECG model provides accurate, clinically interpretable prediction of 1-year mortality and may support risk stratification in secondary care.

Artificial intelligence–enabled electrocardiography (AI-ECG) has emerged as a powerful tool in the diagnosis and management of patients, especially concerning cardiovascular diseases. Most importantly, AI-ECG has shown great promise in allowing early detection of coexisting medical conditions, whether constant, such as significant aortic stenosis, or intermittently evident, as in paroxysmal atrial fibrillation.^1^, ^2^, ^3^, ^4^ AI-ECG is sensitive to small electrocardiographic variations related to biophysical, metabolic, and hormonal alterations that the human eye cannot detect, and with proper training, may detect the risk of disease or medical events long before they are clinically evident.^1^^,^^3^^,^^5^

One of the toughest challenges in patient triage and management is the ability to identify patients who are at high risk of deterioration and death.^6^, ^7^, ^8^ This challenge is even higher in secondary care settings, most commonly in the emergency departments (EDs), where many patients are triaged to hospitalization or discharge, typically with focused assessment and limited resources.^8^ ECG is a ubiquitous and standardized test that is easy and cheap. As a derivative, ECG is performed on most patients requiring health care services, especially in secondary settings, where it serves as a valuable tool for triage.

AI-ECG has demonstrated effectiveness in predicting all-cause mortality among community-dwelling individuals in primary care settings, as well as in critically ill patients and those with specific underlying conditions.^9^, ^10^, ^11^, ^12^ More recently, its application in hospitalized patients to alert clinicians to elevated mortality risk and to predict cardiovascular events and mortality in secondary prevention populations has shown promise, with reported areas under the curve (AUCs) ranging from 0.77 to 0.84.^10^^,^^12^ However, the utility of AI-ECG for predicting out-of-hospital mortality, specifically in secondary prevention settings, remains underexplored. Furthermore, it is still unclear whether incorporating AI analysis of prior ECGs into predictive models can enhance mortality risk stratification in these populations.

This study aimed to construct and validate an AI-ECG model for predicting 1-year all-cause mortality among patients treated in a secondary care setting. We further aimed to explore the components that contribute the most to AI-ECG discrimination.

## Methods

A retrospective population study was performed at Soroka University Medical Center (SUMC), one of the largest referral centers in Clalit Health Services (CHS). CHS is Israel’s largest health care maintenance organization, with over 4.2 million insured citizens, with an extremely low annual turnover of <1%.^13^ As an integral part of CHS, SUMC manages patients using the CHS-integrated electronic health records (EHRs). This comprehensive EHR database has been used in CHS since 2000, storing all patient data from primary, secondary, and tertiary care settings. CHS EHRs are continuously updated with real-time input from administrative, medical, and pharmaceutical systems. This unique, continuously monitored, validated database environment provides high reliability, consistency, and accuracy of exposure and follow-up data. Importantly, all-cause mortality data are updated in CHS EHR immediately as reported in any EHR system, including the Israeli Ministry of Interior, through a distinct algorithm connecting all electronic medical registries in Israel. This study was approved by the Institutional Committee on Human Research of SUMC, Beersheva, Israel.

### Study design

The study population was comprised of adult (age >50 years) CHS members treated in the SUMC emergency medicine department or inpatient services with an electronically documented ECG at their index encounter between January 1, 2014, and December 31, 2019. Data were collected for past documented electronic ECGs before the index encounter. Prior ECGs were considered as such only if they were obtained more than 24 hours from the index encounter ECG. ECGs without complete 12 leads or shorter than 10 seconds were disqualified and not accounted for. Patients in a state of pregnancy or lactation were excluded. The study was approved by the Institutional Review Board of SUMC.

### Study data and covariates

All study covariates were based on information recorded before the index hospital encounter. All medical conditions (based on International Classification of Diseases Version 9 [ICD-9] codes and an internal coding system) were based on records before (up to 6 months) the index hospital encounter.

Background risk factors for all-cause mortality were predefined a priori based on established clinical knowledge and expert consensus by 2 expert clinicians (G.T. and M.H.), rather than selected through data-driven screening procedures. The following 12 variables were included: age, sex, smoking, hypertension, diabetes, dyslipidemia, chronic kidney disease, congestive heart failure, chronic obstructive pulmonary disease, any previous coronary or valvular intervention (a composite of percutaneous coronary intervention, coronary artery bypass surgery, or valvular heart intervention or surgery), use of aspirin, and use of statins. Binary variables were encoded as 0/1 indicators, and age was modeled as a continuous variable.

The time gap between ECG samples was deliberately included as a covariate in the serial AI-ECG models. At each time point, the elapsed time since the prior ECG was incorporated into the feature vector to account for irregular sampling intervals. A multivariable logistic regression model using the predefined clinical covariates was fitted to provide an interpretable baseline comparator. The ECG-derived risk score was concatenated with the predefined clinical covariates to form a structured feature vector at each time point.

Encounter-related diagnoses were defined by the primary diagnosis recorded in the EHR up to 7 days from the index hospital encounter date. The study’s primary outcome was all-cause mortality within 1 year from the index hospital encounter. Given the low number of early events (fewer than 30 patients with 3 qualifying ECGs who experienced mortality within 30 days), the study was not designed to model short-term mortality outcomes.

### AI-ECG training

**Figure 1:**
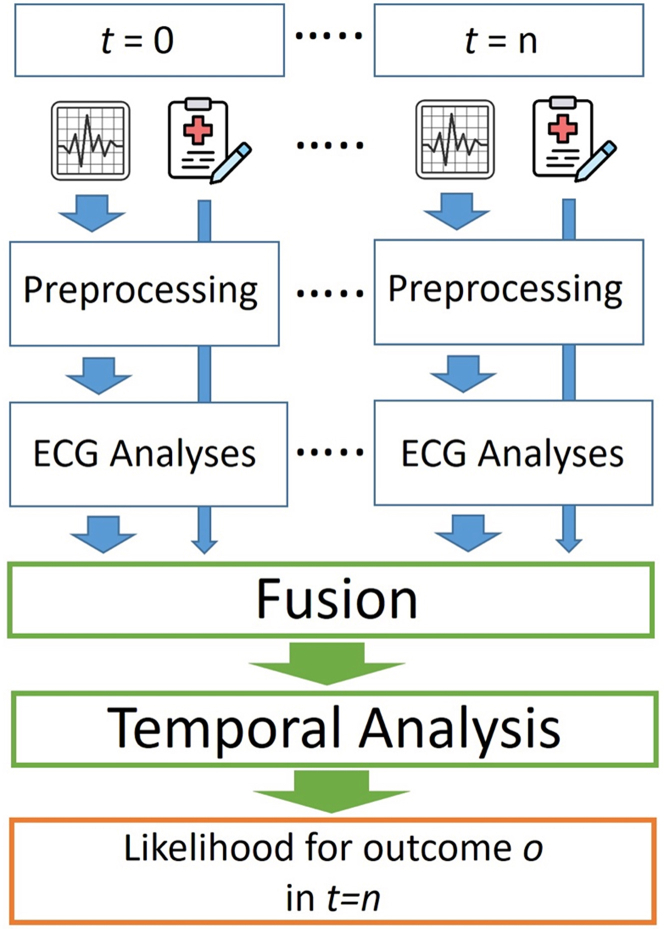
The Phases in the ECG Processing

AI-ECG models were trained based on the following 4 phases (depicted in Figure 1):

1. *ECG preprocessing*: ECG signal cleaning using a 0.5 Hz high-pass Butterworth filter (order = 5), followed by Powerline filtering (powerline = 50).^14^
2. *ECG analysis*: ECG signal analysis was performed using a deep learning model based on a previously validated Residual Network (ResNet) architecture,^15^^,^^16^ with the final layer adapted for prediction of 1-year mortality. The modeling framework was structured in a modular manner, separating per-ECG feature extraction (ResNet) from temporal modeling using a long short-term memory (LSTM) network. Hyperparameters were optimized using randomized search with selection based on validation set performance. The LSTM architecture was derived through iterative expansion from a minimal baseline until validation performance plateaued. The trained network outputs a risk score for each ECG, with optional use of hidden layer representations for higher-dimensional feature extraction.
3. *Concatenation*: Each ECG’s score (or hidden layer) was concatenated with 12 predefined tabular risk factors. In serial models, the time interval between ECGs was additionally included in this vector. This phase's output is a vector representing the ECG exam itself and its context.
4. *Temporal analysis*: For each patient, 3 timestamps (ie, vectors) were fed into the LSTM network.^17^ Missing ECGs were represented using masked values (−1). The time gap between ECG samples was explicitly included as a covariate in the serial AI-ECG models. At each time point, the elapsed time since the prior ECG was incorporated into the feature vector. Each time-step input to the LSTM consisted of the ECG-derived risk score, clinical covariates, and the time interval since the previous ECG. The output of this phase was the likelihood of 1-year mortality for each patient.

The 1-year mortality prediction task was defined as a classification problem, and an ablation study was conducted on the data types (ECG and tabular) and analysis (static and temporal), including comparisons between models with and without explicit temporal encoding.

We divided the patients (not individual ECGs) into train (68%), validation (17%), and test (15%) sets, ensuring that all ECGs from a given patient were assigned to the same partition to prevent information leakage across serial ECGs. Baseline characteristics and outcome rates were comparable across the 3 partitions. For each experiment, we trained the model using the training set, fine-tuned the hyperparameters using the validation set, and conducted the final evaluation using the test set.

### Implementation details

All hyperparameters were optimized using randomized search over predefined parameter spaces, with selection based on validation set performance.

#### Random forest

Scikit-learn's RandomForestClassifier was used for implementation. Hyperparameters (number of estimators, maximum depth, minimum samples per leaf, and minimum samples for split) were chosen using a randomized search.

#### ResNet

A previously published, not pretrained architecture was used.^15^ We changed the original 6-neuron output layer to 2 layers: A Dense layer with 12 neurons with a ReLU activation function and a Dense layer with a single neuron and a sigmoid activation function. We trained the ResNet using the Adam optimizer, a learning rate of 1e-06, batch size of 256, 4,000 epochs with early stopping, and patience of 100 epochs. Hyperparameters were chosen using a randomized search.

#### LSTM

The data were normalized before feeding it to the LSTM. Missing exams were masked with −1. The Neural Net was implemented using Keras, it consists of 6 layers: LSTM (Neurons = 128, Dropout = 0.1), LSTM (Neurons = 64, Dropout = 0.1), LSTM (Neurons = 32, Dropout = 0.1), Dense (16), Dense (8), Dense (1, Activation = Sigmoid). The architecture followed a progressive dimensionality reduction strategy, derived through iterative expansion from a minimal baseline until validation performance plateaued. A dropout rate of 0.1 was selected to balance regularization and preservation of temporal signal.

### Explainability

Model output explanation was performed using SHapley Additive exPlanations, a widely used method to determine the importance of a feature in a machine learning model’s prediction that calculates the contribution of each feature in a prediction by comparing the model’s prediction when the feature is included vs excluded. Explainability of the model’s decision was performed in a 2-step process: 1) 1-year mortality likelihood explanation; and 2) ECG risk score explanation. First, the effect of the ECG risk score and the 12 risk factors on the likelihood of 1-year mortality was plotted, which was followed by examining the influence of the ECG signal on the ECG risk score.

### Statistical analysis

Baseline characteristics of the study population are presented as means and SDs or 95% CIs, or medians and IQR for continuous variables, according to the distribution of the variable of interest. Categorical data are presented as counts and percentages. The Mann-Whitney *U* and chi-square tests were applied to compare the distribution of the variables.

All measures of performance were based on the test set data. Survival rates over time were explored using life tables and presented using Kaplan-Meier curves. The receiver-operating characteristic curve was used to assess model discrimination based on predicted probabilities of 1-year mortality. Calibration was assessed by comparing predicted probabilities with observed event rates using calibration plots based on deciles of predicted risk. Overall probabilistic accuracy was quantified using the Brier score. Calibration slope and intercept were estimated by regressing observed outcomes on the logit of predicted probabilities. Expected calibration error was calculated to summarize absolute deviations across risk strata. To define a binary classification operating point, the threshold for a positive screen was selected on the validation set by maximizing the F1-score, which balances precision and recall and is well suited to imbalanced outcomes. The selected threshold was then applied without modification to the independent test set to compute sensitivity, specificity, accuracy, precision, and other threshold-dependent performance measures. The diagnostic OR, defined as the ratio of the positive likelihood ratio to the negative likelihood ratio, and its corresponding 95% CI were also calculated. A multivariable logistic regression model was additionally fitted using the predefined clinical covariates as an interpretable baseline comparator. The final threshold selected on the validation set was 0.24.

To assess model stability and variability, a nonparametric bootstrapping analysis (1,000 iterations with replacement) was performed on the test set to derive 95% CIs for performance metrics. This approach provides an estimate of performance variability across resampled data sets, supporting the stability of the model. Model convergence and potential overfitting were evaluated using training and validation loss curves (Supplemental Figure 1).

For all analyses, significance was set at a 2-sided *P* value of ≤0.05. Statistical analyses were performed using R: Core Team, statistical software version 4.1.2 (R Foundation for Statistical Computing), RStudio Team (2020). RStudio: Integrated Development for R. RStudio, PBC, URL http://www.rstudio.com/.

## Results

### Study population

**Figure 2:**
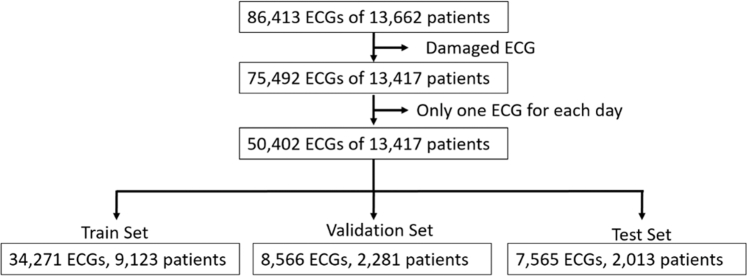
Study Flowchart A total of 13,662 patients were eligible for inclusion. After excluding patients with technically faulted ECG recordings, 13,417 remained and comprised the final study population. After excluding multiple ECGs per day, a total of 50,402 were available for analysis. Patients were divided into train (68%), validation (17%), and test (15%) subsets.

During the study period, 13,662 patients had a hospital encounter where an ECG was obtained with at least 2 documented prior ECGs. After excluding patients with technically faulted or incomplete ECGs at any time point, 13,417 patients remained and comprised the final study population. The study population was divided into train (n = 9,123 [68%]), validation (n = 2,281 [17%]), and test (n = 2,013 [15%]) data sets. The study flowchart is depicted in Figure 2.

The most common index ECG acquisition place was at the ED (68.1%). Cardiac or respiratory diagnoses comprised 34.7% of the attributed clinical diagnoses within 7 days of the reference ECG. A total of 1,260 (9.7%) patients had no documented diagnosis that could be attributed to their reference encounter, where the reference ECG was obtained.

The median time difference between the index encounter ECG and the previous ECG was 1.3 (0.1-8.8) months and 3.2 (0.1-14.5) months, for the closest and farthest ECGs, respectively.

### Baseline characteristics

The baseline characteristics of the final study population were similar across the modeling data sets and are presented in Table 1. Patients had a median age of 69 (IQR: 57-80), and 55% were males. At baseline, the patients had a relatively high burden of cardiovascular and metabolic comorbidities, with diabetes, hypertension, dyslipidemia, and ischemic heart disease rates ranging from 40% to 65%. Furthermore, ∼20% of the patients had been diagnosed with congestive heart failure or chronic kidney failure at baseline. Congruently, nearly 30% of the patients had previous coronary intervention (percutaneous coronary artery intervention or coronary artery bypass surgery). Baseline patient characteristics were balanced across the training, validation, and test data sets.

**Table 1:** Study Population Characteristics Across Modeling Data Sets

	Total (N = 13,417)	Training (n = 9,123)	Validation (n = 2,281)	Test (n = 2,013)
Age (y)	69 (57-80)	69 (57-80)	69 (57-81)	70 (57-80)
Male, %	7,374 (55)	4,979 (55)	1,294 (57)	1,101 (55)
Smoking, %	5,228 (39)	3,577 (39)	910 (40)	741 (37)
BMI, kg/m^2^	26.7 (23.4-30.9)	26.6 (23.4-30.8)	26.9 (23.4-30.8)	26.8 (23.4-31.0)
CCI, median (IQR)	5 (3-8)	5 (3-8)	5 (3-8)	5 (3-8)
Hypertension, %	8,421 (63)	5,667 (62)	1,456 (64)	1,298 (65)
Diabetes, %	5,379 (40)	3,642 (40)	912 (40)	825 (41)
Dyslipidemia, %	8,106 (60)	5,461 (60)	1,398 (61)	1,247 (62)
Chronic liver disease, %	845 (6.3)	553 (6.1)	155 (6.8)	137 (6.8)
CKD, %	2,737 (20)	1827 (20)	496 (22)	414 (21)
IHD, %	6,164 (46)	4,172 (46)	1,049 (46)	943 (47)
CHF, %	2,963 (22)	2007 (22)	522 (23)	434 (22)
COPD, %	1825 (14)	1,201 (13)	344 (15)	280 (14)
Asthma, %	1,242 (9.3)	817 (9.0)	222 (9.7)	203 (10.1)
Cancer, %	2,577 (19)	1752 (19)	456 (20)	369 (18)
CABG, %	1,218 (9.1)	811 (8.9)	216 (9.5)	191 (9.5)
PCI, %	2,455 (18)	1,648 (18)	442 (19)	365 (18)
Valvular heart surgery, %	513 (3.8)	353 (3.9)	90 (3.9)	70 (3.5)
Aspirin, %	3,295 (25)	2,230 (24)	598 (26)	467 (23)
Statins, %	4,110 (31)	2,774 (30)	712 (31)	624 (31)
P2Y12 inhibitors, %	1,460 (11)	948 (10)	288 (13)	224 (11)
DOAC, %	1,445 (11)	991 (11)	252 (11)	202 (10)

Baseline diagnoses, procedures, and medications were considered if documented at least 7 days before the index encounter ECG.

BMI = body mass index; CABG = coronary artery bypass surgery; CCI = Charlson's Comorbidity Index; CHF = congestive heart failure; CKD = chronic kidney disease; COPD = chronic obstructive pulmonary disease; DOAC = direct oral anticoagulants; IHD = ischemic heart disease; PCI = percutaneous coronary artery intervention.

### Mortality rates

During a median follow-up of 1.5 years (IQR: 0.5-3.8 years), a total of 4,372 (34%) of the patients died. The median time to death was 99 days (IQR: 19-430 days). Most death cases (74% of all deaths) occurred within 1 year of the index encounter. The number of patients with 3 qualifying ECGs who experienced mortality within 30 days was limited (n < 30; 0.6% of all deaths). Survival rates were similar across the training, validation, and test sets (Supplemental Figure 2).

### One-year mortality prediction

**Figure 3:**
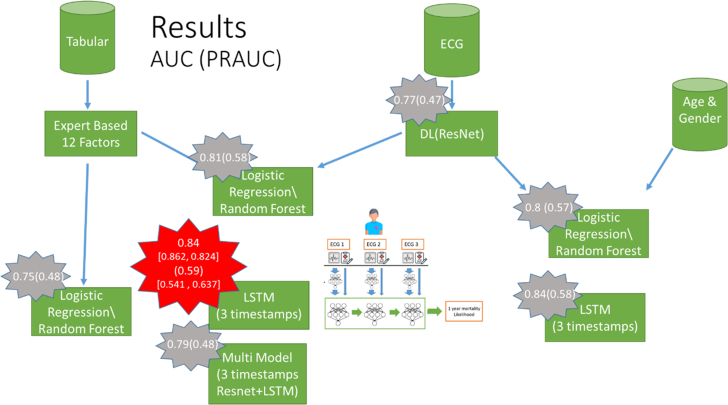
One-Year Mortality Modeling Approach To determine the optimal model for predicting 1-year mortality, we compared and integrated models using clinical data (tabular), a single index ECG, and up to 3 ECGs (including the index and up to 2 prior ECGs). The ECG-based models demonstrated higher AUCs compared to those based solely on clinical data. The best-performing model included 3 ECG timestamps, age, and sex. Further inclusion of additional clinical risk factors did not improve the model’s performance. AUCs = areas under the curve; LSTM = long short-term memory; ResNet = Residual Network.

The modeling approach to predict 1-year mortality was based on the comparison and integration of clinical data and single or multiple ECGs (Figure 3). First, we constructed a baseline model based on predefined clinical variables to predict 1-year mortality. A multivariable logistic regression model (Supplemental Table 1) using these covariates demonstrated that several factors were independently associated with mortality, including congestive heart failure, chronic kidney disease, chronic obstructive pulmonary disease, and diabetes mellitus, while prior coronary or valvular intervention and statin use were associated with lower risk. The clinical prediction model yielded an AUC of 0.75 to predict 1-year mortality.

Next, we trained an independent machine learning algorithm to detect 1-year mortality risk by the single index encounter AI-ECG (the most recent ECG). The single-AI-ECG models achieved an AUC of 0.77 for predicting 1-year mortality. Further inclusion of age and sex in the AI-ECG model resulted in an AUC of 0.8. However, further inclusion of all 12 clinical risk factors resulted in a modest increase in AUC to 0.81.

**Figure 4:**
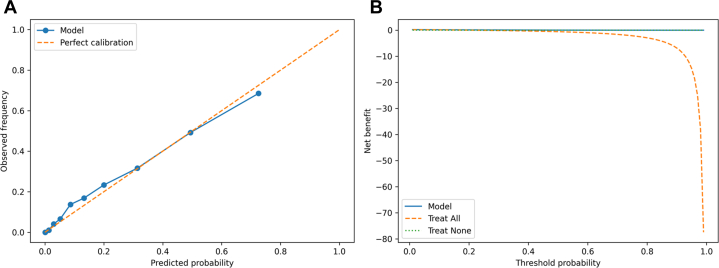
Calibration and Clinical Utility of the Serial AI-ECG Model (A) Calibration plot comparing predicted probabilities with observed event rates across deciles of risk. The dashed line represents perfect calibration. (B) Decision-curve analysis showing the net benefit of the model across a range of threshold probabilities, compared with treat-all and treat-none strategies.

In the final step, we provided the prediction model with 2 previous AI-ECG timestamps, in addition to the index single AI-ECG, along with age and sex. This model yielded an AUC of 0.84. Inclusion of all clinical risk factors did not result in a change in the AUC (0.84). Model performance was evaluated using an independent test set, and variability was assessed using nonparametric bootstrapping, yielding a 95% CI for the AUC of 0.824 to 0.862. The narrow CI indicates low variability and stable model performance across resampled cohorts. Calibration analysis demonstrated good agreement between predicted and observed risks (Figure 4A). The calibration plot showed close alignment across most risk strata, with minor deviations at higher predicted probabilities. The Brier score was 0.123. The calibration slope was 0.87 (95% CI: 0.77-0.97) and the intercept was −0.03 (95% CI: −0.22 to 0.12), indicating mild overfitting without substantial systematic bias. The expected calibration error was 0.030. Decision-curve analysis demonstrated that the model provided greater net benefit than both treat-all and treat-none strategies across a range of clinically relevant threshold probabilities (Figure 4B). The optimal classification threshold, determined on the validation set by maximizing the F1-score, was 0.24. When applied unchanged to the independent test set, this threshold yielded a sensitivity of 70%, a specificity of 80%, and a positive predictive value of 49%. This operating point reflects a balance between identifying patients at risk of mortality and limiting false positive classifications. As expected, threshold-dependent metrics on the test set differed modestly from those observed during validation, reflecting generalization to unseen data. Removal of temporal interval information in ablation analysis reduced model performance to an AUC of 0.81, compared with 0.84 in the full model.

### Saliency (explainability)

**Figure 5:**
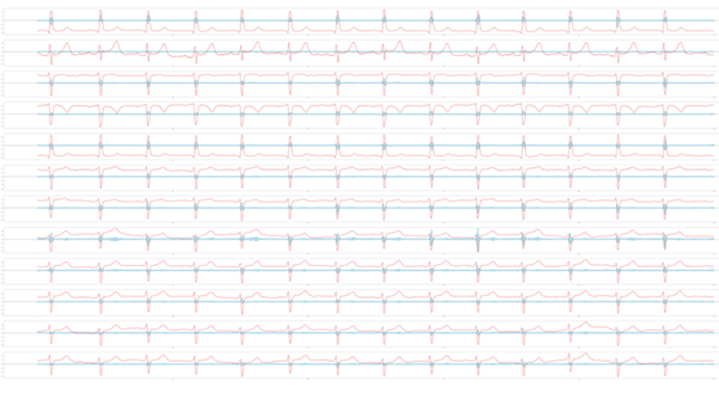
Saliency Map A saliency map on a representative ECG. The artificial intelligence model prediction was mostly based on the QRS complexes in all lead tracings and specifically the TP segment of the V_2_-V_3_ precordial lead tracings.

A saliency map on a representative 12-lead AI-ECG is shown in Figure 5. Interestingly, the model’s prediction was mostly based on the QRS complexes in all lead tracings and specifically the TP segment of V_2_-V_3_ precordial lead tracings. This may imply that, unlike clinically expected, the QT segments are less predictive of mortality risk than QRS complex changes. However, repolarization changes in lead V_2_-V_3_ may be more related to mortality risk among high-risk patients, as in this study.

## Discussion

This study, conducted among over 13,500 adult patients treated in the ED or inpatient units at a large tertiary medical center, aimed to evaluate whether AI-ECG analysis, based on the index ECG alone or in combination with prior ECGs, could enhance mortality risk stratification up to 1 year after presentation (Central Illustration). The findings demonstrated that an AI model incorporating the index ECG, 2 prior ECGs, age, and sex outperformed models based solely on clinical exposure variables or a single AI-ECG from the index encounter. Both single- and multi-time point AI-ECG models provided superior prognostic value compared to clinical variables alone. Furthermore, adding additional clinical variables to the model that already included AI-ECG data, age, and sex did not improve predictive performance, highlighting the independent value of AI-ECG for mortality prediction.

**Central Illustration:**
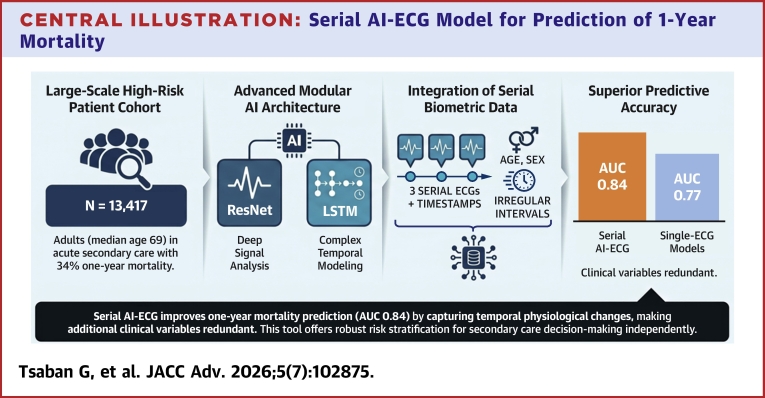
Serial AI-ECG Model for Prediction of 1-Year Mortality Overview of the study design and key findings. A deep-learning model using single or serial ECGs (index and 2 prior) was developed to predict 1-year mortality in a secondary care population. The serial AI-ECG model achieved improved performance (AUC: 0.84), with good calibration and clinical utility, supporting its potential role in risk stratification. Abbreviations as in Figure 3.

Previous studies have shown that AI-ECG models, whether trained to detect mortality risk directly or to identify underlying pathophysiology such as diastolic dysfunction, perform well across various clinical settings.^9^, ^10^, ^11^, ^12^^,^^18^ This study focused on patients presenting to the hospital with acute symptoms or illness, representing a uniquely high-risk and heterogeneous population. Prognostication in this group is challenging due to the variability in presentation and underlying conditions. Simple, reproducible, and cost-effective tools for short- and intermediate-term risk stratification in this setting could significantly impact care planning, resource allocation, and follow-up.

This study shows that an AI-ECG–based model provides stronger prognostic performance than clinical variables alone. The addition of prior ECGs further improved performance, suggesting that longitudinal ECG data capture clinically meaningful changes over time rather than simply increasing the amount of input information. This may be particularly relevant in secondary prevention settings, where patients frequently undergo repeated evaluations. To our knowledge, the use of multiple ECGs in this way for mortality prediction has not been previously described.

The limited incremental value of additional clinical variables beyond age and sex likely reflects the ability of the ECG signal to capture downstream physiological effects of these risk factors. This aligns with prior studies showing that AI-ECG models encode prognostic information not fully represented by conventional clinical variables. Model performance was evaluated at a predefined operating threshold selected on the validation set and applied unchanged to the independent test set. At this operating point, the model achieved a balanced tradeoff between sensitivity and specificity, supporting its use for risk stratification rather than relying on discrimination metrics alone. Although the study was designed as a classification task focused on 1-year mortality, time-to-event approaches such as Cox models or neural network–based survival methods could offer additional flexibility by incorporating time-to-event information and censoring. In this cohort, follow-up for mortality was complete and most events occurred within 1 year, which supports the use of a fixed-horizon outcome. The classification framework also provides risk estimates that are straightforward to interpret in clinical practice. The model also demonstrated good calibration, with predicted risks closely aligned with observed event rates across most of the risk spectrum. Mild deviations at higher predicted probabilities suggest some degree of overestimation in high-risk patients, although overall agreement remained strong. These findings support the clinical interpretability of the model’s predicted probabilities and their potential use in risk stratification. Future work may extend this approach to better characterize longer-term risk trajectories.

Despite the smaller sample size compared with some previously published analyses, the use of an LSTM framework incorporating 3 ECG time points allowed us to achieve comparable performance. The combination of a validated architecture, modular design, and stable performance across resampled data sets supports the robustness of the model. By leveraging longitudinal ECG data, the model captures within-patient trajectories that may not be evident from a single time point. External validation will be important to further assess generalizability.

Saliency map analysis revealed that the model primarily focused on the QRS complex and TP segment in precordial leads V_2_ and V_3_, rather than the traditionally emphasized intervals like the QT segment or T-wave. The QRS complex, particularly its morphology and duration, is a well-established marker of cardiovascular risk.^19^ Interestingly, the TP segment, often considered electrically neutral, emerged as an unexpected yet consistent contributor to the model’s predictive performance. This finding aligns with previous explainability analyses in AI-ECG models, such as the one trained to detect aortic stenosis.^2^ These results suggest that significant mortality risk may be hidden within ECG segments not typically associated with common cardiac pathologies. Future research is needed to better understand the relationship between these less conventional ECG segments and patient outcomes.

### Study limitations

Several limitations should be acknowledged. First, although the sample size was substantial, the data were drawn from a single tertiary center, which may limit generalizability. Second, the clinical model was based on documented exposure variables, and as with all retrospective analyses, unmeasured confounders may have influenced outcomes. Third, the limited number of early mortality events (n < 30 within 30 days among patients with 3 qualifying ECGs) precluded robust modeling of short-term outcomes. Accordingly, the model’s predictive performance is most representative of longer-term mortality risk (up to 1 year). While the model captures physiological signals associated with survival, the low prevalence of early events limits the ability to distinguish between immediate and longer-term risk. Future studies with larger numbers of early events are needed to evaluate short-term prediction. Fourth, the timing of prior ECGs varied, although the median interval was relatively short (just over 1 month for the closest ECG, and just over 3 months for the most remote), reflecting common clinical practice in secondary prevention. Lastly, the model predicted all-cause mortality among a broad hospital-presenting population rather than within a specific disease group. While this could be seen as a limitation, this may be considered as a strength, as it reflects a real-world, undifferentiated population where such tools are most needed. At the selected operating threshold, derived on the validation set and applied to the independent test set, the model achieved a sensitivity of 70%, a specificity of 80%, and a positive predictive value of 49%. These results reflect a balanced tradeoff between identifying patients at elevated risk and limiting false positive classifications. However, the moderate positive predictive value indicates that a proportion of patients identified as high risk would not experience the outcome, which should be considered when interpreting the model in clinical practice. The optimal operating point may vary depending on the intended clinical use and the relative importance of false negative and false positive predictions.

## Conclusions

This study presents a model for predicting all-cause mortality in secondary prevention settings, using 3 ECGs from the index encounter and 2 prior ECGs. The model, incorporating these 3 ECG timestamps along with age and sex, achieved an AUC of 0.84, outperforming models based on clinical variables or a single ECG. These findings underscore the potential of AI-ECG analysis, particularly when combined with longitudinal ECG data and demographic variables, as a valuable tool for predicting 1-year mortality. While promising, further external validation and refinement are needed before widespread clinical implementation. As AI continues to evolve, such models may enhance personalized medicine, supporting clinicians in improving patient outcomes. However, successful integration into clinical practice will require validation across diverse health care settings with varying patient demographics and resources.

## Funding support and author disclosures

The authors have reported that they have no relationships relevant to the contents of this paper to disclose.
