# Supplementary material for: Artificial Intelligence–Enabled Serial Electrocardiograms for Prediction of All-Cause Mortality in Secondary Care Settings
Source: JACC Adv. 2026 Jun 17;5(7):102875. doi: 10.1016/j.jacadv.2026.102875 (PMC13292797; doi:10.1016/j.jacadv.2026.102875)
Supplement: Supplemental Material [file mmc1.pdf]

----Supplementary data----

Artificial intelligence-enabled serial electrocardiograms for prediction of all-cause mortality in secondary care settings

Gal Tsaban MD PhD[1,2*], Asaf Harari PhD[4, 2*], Adi Shiloh MD[3], David Shamia MD[1], Lior Rokach PhD[4], Michal Gordon PhD[3], Moti Haim MD[1,2†], Gilad Katz PhD[4†]

[1]Cardiology Department, Soroka University Medical Center, Beersheva, Israel
[2]Faculty of Health Sciences, Ben Gurion University of the Negev, Beersheva, Israel
[3]Clinical Research Center, Soroka University Medical Center, Beersheva, Israel
[4]Faculty of Computer and Information Science, Ben Gurion University of the Negev, Beersheva, Israel
* The first two authors contributed equally to the study
†The last two authors are equal contributors

Contents

Supplement 1: Training and validation loss curves demonstrating model convergence 2

Supplement 2: Survival across the training, validation, and test data sets 3

Supplement 3: Multivariable logistic regression model for 1-year mortality 4

----Supplementary data----

Supplement 1: Training and validation loss curves demonstrating model convergence

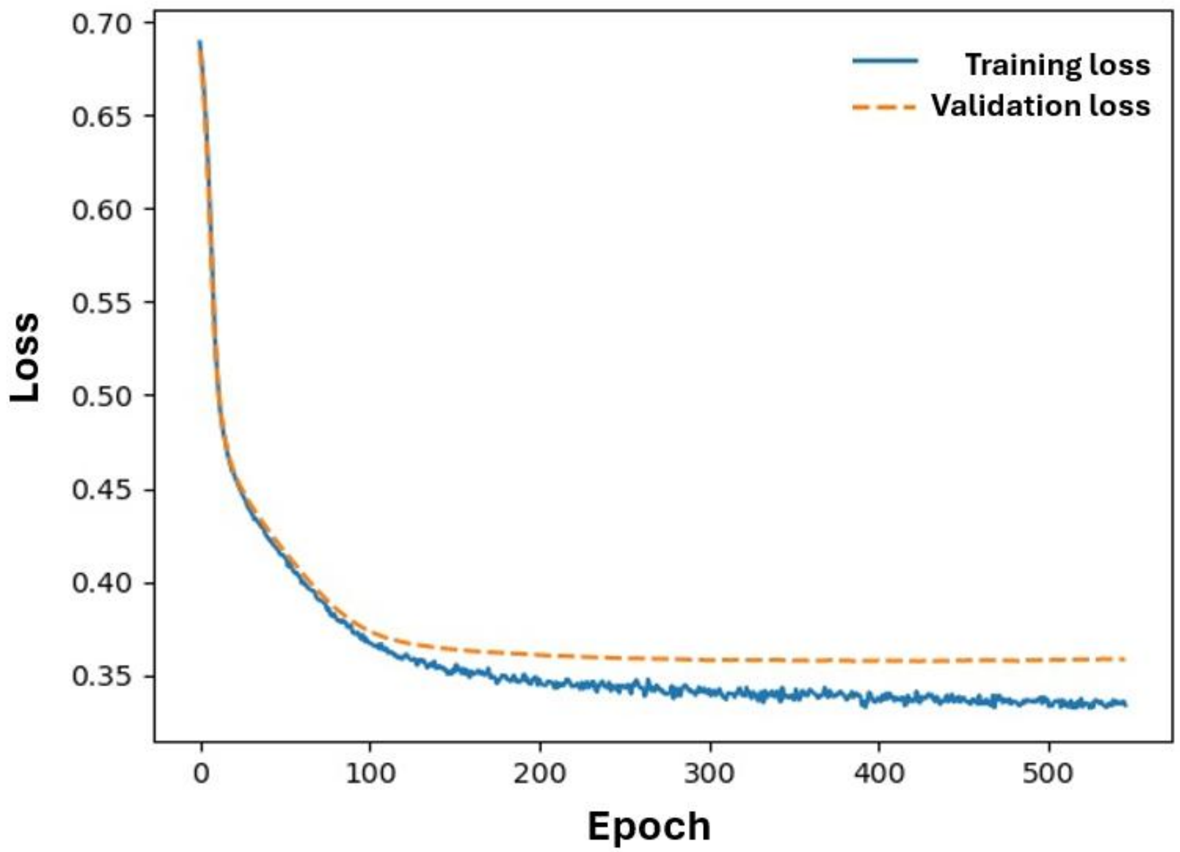

----Supplementary data----

Supplement 2: Survival across the training, validation, and test data sets

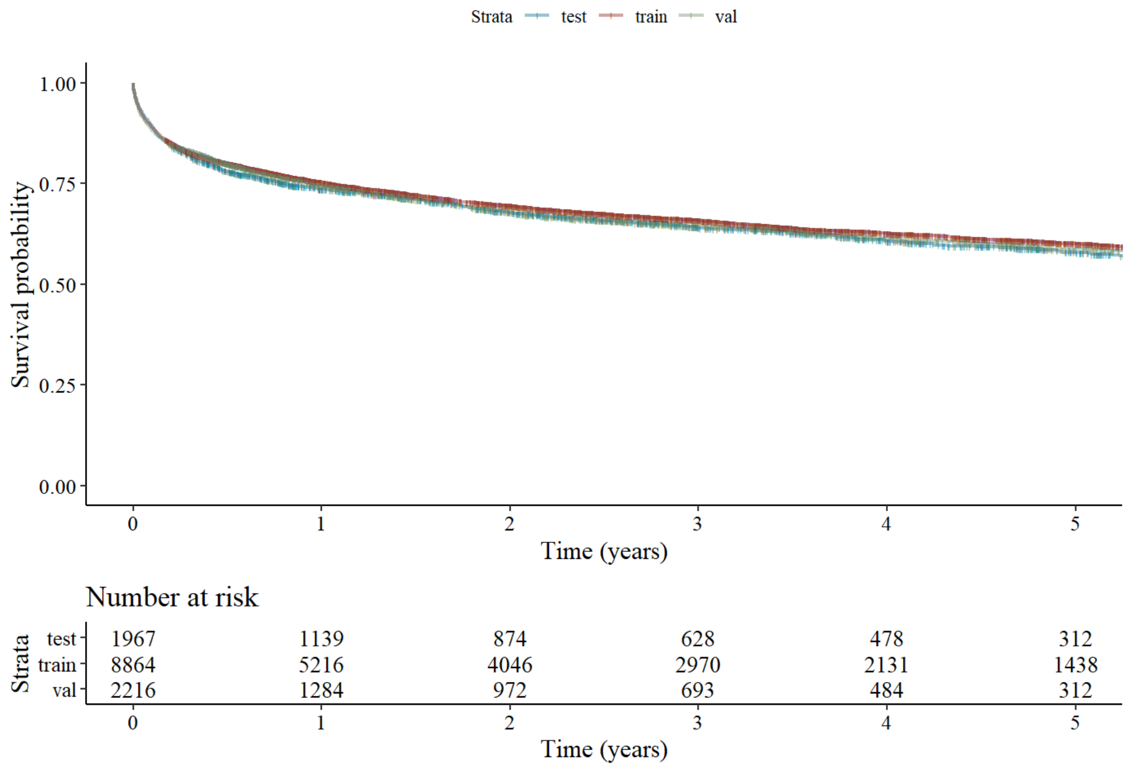

----Supplementary data----

Supplement 3: Multivariable logistic regression model for 1-year mortality

	Odds Ratio (95% CI)	P-value
Age	1.06 (1.06-1.07)	<0.001
Male sex	1.02 (0.91-1.14)	0.800
Smoking	1.17 (1.02-1.35)	0.024
HTN	0.93 (0.81-1.07)	0.300
Diabetes mellitus	1.26 (1.11-1.42)	<0.001
Dyslipidemia	0.87 (0.76-0.99)	0.035
CKD	1.38 (1.19-1.59)	<0.001
CHF	1.52 (1.30-1.76)	<0.001
COPD	1.34 (1.15-1.56)	<0.001
Past PCI, CABG or valvular heart surgery	0.59 (0.49-0.69)	<0.001
Aspirin (chronic medication)	0.88 (0.76-1.01)	0.068
Statins (chronic medication)	0.80 (0.70-0.92)	0.002

AUC = 0.74
